# Aerobic and Anaerobic Fitness according to High-Intensity Interval Training Frequency in Youth Soccer Players in the Last Stage of Rehabilitation

**DOI:** 10.3390/ijerph192315573

**Published:** 2022-11-23

**Authors:** Shuren Yan, Yonghwan Kim, Yongchul Choi

**Affiliations:** 1 Department of Physical Education, Luoyang Normal University, Luoyang 471934, China; 2 Department of Physical Education, Gangneung-Wonju National University, Gangneung 25457, Republic of Korea

**Keywords:** youth soccer players, high intensity interval training, VO_2_peak, anaerobic power, isokinetic strength, body composition, training frequency

## Abstract

In the last stage of rehabilitation, high-intensity interval training (HIIT) for improving physical fitness is appropriate for return-to-play; however, some youth athletes visit the rehabilitation center less frequently due to conflict with their distance to center, and academic schedule. We tested the effects of short-term low-frequency HIIT in 54 youth male soccer players, after dividing them into a low-frequency group (LFG, *n* = 27 players) and a high-frequency group (HFG, *n* = 27 players). Muscle mass and body fat were measured using a body composition test, and VO_2_peak and exercise duration were measured using a treadmill. Five sets of anaerobic peak power and fatigue were measured repeatedly using the Wingate test. To evaluate knee joint muscle function, 60°/s, 180°/s, and 240°/s were measured using the isokinetic muscle function equipment. HIIT sessions were conducted twice a week for LFG and five times a week for HFG for 4 weeks. In this study, Wilcoxon signed-rank test and Mann–Whitney U test were mainly used for analysis. Significant improvements in VO_2_peak, anaerobic peak power, and knee strength were observed after intervention in both groups (*p* < 0.05). In the post test, there were significant differences between groups in VO_2_peak (LFG, 56.4 vs. HFG, 57.1 mL/kg/min; *p* = 0.035), exercise duration (LFG, 972.3 vs. HFG, 990.4 s; *p* = 0.041), Wingate anaerobic peak power 5 sets (LFG, 606.3 vs. HFG, 629.3 Watt; *p* = 0.039), and muscle function test 240°/s (LFG, 68.5 vs. HFG, 70.2 Jouls; *p* = 0.010). However, neither group showed significant changes in body composition, such as muscle mass or body fat (*p* > 0.05). In conclusion, although it is a short-term training, the effect of HIIT was shown in the HFG as well as LFG. Although HFG improved physical fitness, significant improvement was also achieved in LFG. Therefore, in the last stage of rehabilitation, low frequency as well as high frequency HIIT would be an appropriate training method to improve physical fitness for youth soccer players.

## 1. Introduction

Soccer is an intense sport, and the risk of injury increases during play and practice because of frequent physical contact with opponents and running, jumping, and cutting [1]. Depending on the severity of the injury, minor injuries may heal with rest; however, some injured players are inevitably removed from the team and go on a rehabilitation or recovery course. Many injured athletes experience decreased fitness because of detraining during this period. According to a detraining study, triathletes who stopped training for 30 days had a 4.7% reduction in their maximum aerobic capacity [2]. In another study for 5-week detraining swimmers, the VO_2_peak reduced from 46.7 mL/kg/min to 43.1 mL/kg/min, and accompanied by an increase of 1.8 kg body fat [3].

However, to return to sports after rehabilitation, restoring physical fitness to the pre-injury level is necessary. Soccer is a high-intensity sport that involves walking or running about 8 to 12 km in a 90 min game duration. Although most of the game uses aerobic energy, elite soccer players require high aerobic and anaerobic fitness levels because an intermittent high-intensity activity is often required [4,5,6].

High-intensity interval training (HIIT) is known to improve speed, power, and aerobic endurance more effectively than moderate continuous training (MCT) [7,8,9]. HIIT is considered one of the most effective forms of exercise for improving endurance and tolerance to anaerobic exercise as a training method to reduce overall weekly exercise and increase intensity [10,11]. HIIT is reportedly effective when performed for three sessions per week for at least six weeks to reduce the negative reaction phenomenon caused by glycogen depletion, accumulation of metabolites in the muscle, and neuromuscular tension and to improve endurance and athletic performance [12,13].

The total exercise volume, intensity and duration, and recovery time between exercises should be wholly considered when planning HIIT [14]. HIIT design should consider the total exercise volume, exercise intensity and duration, and recovery time between exercises. A model with a shorter duration, similar to a play pattern, is suitable for soccer players [15,16]. The exercise intensity of this model lasts 12 to 15 s at 90% of the maximum heart rate, and it is effective for physiological stimulation to be performed approximately 15 times per set. The recovery time between workouts is 2–3 times longer than the workout time [17,18,19].

Therefore, it is necessary to pay attention to the effects of a comprehensive train-ing program on the amount or frequency of HIIT training suitable for youth athletes. Well structured program is essential to improve the performance of elite athletes and avoid excessive muscle fatigue. Inadequate training load and recovery can be stressful for athletes and can reduce their performance [20]. Although many studies conducted mid- to long-term experiments of more than 6 weeks to confirm the effect of HIIT intervention, and most reported positive results [21,22], some reported positive results even in studies of 4 weeks duration or less [23,24,25].

However, most short-term HIIT intervention studies have been conducted on adult athletes, and studies on adolescent or youth athletes are rare. Particularly, in the case of youth elite athletes, the maintaining frequent rehabilitation training center visits is difficult because they have to honor their academic commitments [26,27]. This study was conducted to confirm the effects of low-frequency and high-frequency HIIT per week on youth soccer players who have fully recovered their strength and function and are in their last stage of rehabilitation. Our research could provide valuable information to coaches and sports scientists to better understand the physiological adaptation and performance benefits of HIIT for youth soccer players and to improve training program outcomes.

## 2. Methods

### 2.1. Experimental Design

After registration and identification, the participants signed a written informed consent form. They were checked by specialists for clinically significant cardiovascular, neurological, and other orthopedic conditions. Additionally, psychiatric discomforts such as depression and anxiety were confirmed by a specialist. If the above tests were normal, they were enrolled in this study. Since the variables in this study were physical fitness tests, stretching, running, and cycling were performed for 20–30 min for adequate warm-up. Body composition, isokinetic muscle strength test, Wingate anaerobic power, and graded treadmill exercise tests were performed in that order. Sufficient rest was allowed between each test. For body composition analysis, alcohol and caffeine were restricted for 8 h before the test, and there were no strict restrictions on food and drink during the study period. At the end of the test, the patient returned home after being checked for abnormalities. These tests were performed pre and post the intervention. The HIIT program was conducted for 4 weeks: the low-frequency group (LFG) participated in two sessions per week (total of eight sessions), and the high-frequency group (HFG) participated in five sessions per week (total of 20 sessions). The group assignments were made by considering the individual circumstances of the participants such as distance to center, time, and school academic course.

### 2.2. Participants

The appropriate sample size required for the study was calculated using the G*power software (G*power 3.1, University of Düsseldorf, Düsseldorf, Germany). The settings were as follows: F test and repeated measures, within-between interaction, effect size f = 0.25, α error = 0.05, power (1-β err prob) = 0.95, number of measurements = 2, number of groups = 2, and corr among rep measures = 0.5. The calculated results included 54 samples.

This study was conducted with 54 male high school soccer players (27 each in the LFG and HFG) who visited a rehabilitation center with injuries. The conditions for inclusion were players who could not participate in team training for more than four weeks due to injury. After undergoing rehabilitation and conditioning courses, their range of motion and muscle strength were restored, and there was almost no pain. As the last stage of rehabilitation, it corresponds to the stage of improving performance for return-to-play. The exclusion criteria were elbow and shoulder injuries and those who underwent or were planned to undergo surgery. This study complied with the Declaration of Helsinki for ethical research, and written consent was obtained from the players, guardians, or team coaches. This study was approved by the institutional review board of Gangneung-Wonju National University (2021-11).

### 2.3. Graded Exercise Test

The maximum aerobic exercise capacity test was conducted using a Quark CPET treadmill (Cosmed Co., Rome, Italy). Before conducting the graded exercise test (GXT), the participants were asked about their experience with a treadmill. Inexperienced individuals practiced on a treadmill at low to high speeds. To measure the VO_2_ value in the treadmill-based test, a mask, hose, and sensor were used to analyze the gas exhaled from the mouth and nose. Absolute or relative test termination criteria were applied according to the American College of Sports Medicine (ACSM) guidelines [28]. Simultaneous electrocardiogram (ECG) measurements were performed to confirm the safety of the heart and heart rates. The speed and slope were gradually increased every 3 min, according to the Bruce protocol. Rated perceived exertion (RPE), heart rate, and blood pressure were recorded at each step of the test, and test termination was performed on an all-out basis until the participant’s request. In the all-out state, the exercise was continued until the participant exhausted his or her full force. The participant’s RPE scale of 17 or higher or a respiratory exchange rate of 1.15 was set as the endpoint.

The O_2_ value was monitored in real-time using a breath-by-breath method, and the record was displayed as an average of 20 s. Using the result sheet, a researcher determined the anaerobic threshold (AT) value. The AT value is the point where the values of ventilation, O_2_, and CO_2_ increase suddenly; the later it appears, the higher the fitness. This study analyzed VO_2_peak, AT, AT heart rate (ATHR), all-out exercise time, and heart rate at 1 min of recovery.

### 2.4. Wingate Test

The Wingate test used to measure anaerobic power was performed using a friction-loaded bicycle ergometer (Monark Model 864 Crescent AB, Varberg, Sweden). Before the test, participants practiced with sufficient instruction from the examiners to familiarize themselves with the test equipment and methods. The inspection protocol provided by Bar-Or et al. [29] was applied, and all tests were supervised by the same investigator. Because the Wingate test is the maximum ability test, 60, 90, 120, and 150 revolutions per min (RPM) paddling was practiced before the test, and the load was also experienced at 1, 2, and 3 KP.

In each test, the participant sat on a cycle ergometer and adjusted the saddle height, foot position of the pedals, and comfortable upper body position. The optimal riding posture was the same throughout the study. After adequate practice, a 10 min recovery time was allowed. The actual test time was 30 s, and the participants were instructed to maintain the maximum pedaling speed. They received oral encouragement during the test, and their pedaling speed was recorded every 5 s during sprint cycling. When the 30-s test was completed, a 2 min break was allowed; subsequently, a retest was performed. A total of five sets were performed, and records were analyzed for one, three, and five sets in the study. The Wingate test load was weight multiplied by 0.075 kg. The peak power (watt), peak power per body weight (watt), and fatigue index (%) were calculated. The higher the peak power and peak power per body weight, the higher the fitness, and the lower the fatigue index, the superior the sustaining ability.

### 2.5. Isokinetic Strength Test

Knee flexion and extension strengths were measured using an isokinetic dynamometer (Humac Norm, CSMi, Stoughton, MA, USA). The test was set to uniaxial muscle contraction and was performed in three modes: maximum strength, 60°/s (Nm/kg); average power, 180°/s (average power/kg); and endurance, 240°/s (total Joules) [30].

The isoproperty test measures knee flexion, extension, and posture while sitting on a chair. The axis of rotation of the dynamometer arm was set to coincide with that of the lateral femur of the test knee. The examiner set the setting between 0–90°, controlled by a computer. The possibility of an accident was prevented by attaching a safety pin to the dynamometer. The thighs, pelvis, and torso were secured to the examination chair using Velcro belts to prevent negative body movements that could affect the accuracy. To get accustomed to the isokinetic contraction rate, the participants performed several repetitions at various angular velocities. To examine the posture, the participants first examined the preferred leg and positioned the knee at 90°. Simultaneously, the knee was extended to 0°, and the flexion force was measured by performing flexion up to 90° at the next signal. The participants were instructed to contract to the maximum possible extent and maintain contraction throughout the range of motion. For the test, three rehearsals and four repetitions at 60°/s, four repetitions at 180°/s, and 25 repetitions at 240°/s were measured. Between each angular velocity, the participants rested for at least 2–3 min. When the examination was completed for one leg, the examination of the other leg was performed after adequate rest. In the analysis, flexion and extension were combined, and the value per body weight was used.

### 2.6. Body Composition

Body composition was measured using bioelectrical impedance Inbody 770 (Inbody Inc., Seoul, Republic of Korea). Each participant took off their shoes and socks and was examined while standing on the equipment. The contact surfaces of the electrodes on the machine were the forefoot, heel, thumb, and palm. Alcohol was used to clean the skin of the right foot and the foot where the electrode was touched. The knees and elbows were straightened, and the arms and legs were spread apart so that the skin did not overlap.

The test was performed after the participants had passed urine. Although there was no restriction on food and drink, the test was performed after an 8 h fast which was the adequate time required for complete digestion. This was done to eliminate the possibility of any effect on performance caused by the presence of food in the intestines after overeating or drinking. Fat and muscle mass were indicated on the result sheet, and body fat and muscle variables were used as absolute (kg) and relative values (absolute value/weight) × 100 in the analysis.

### 2.7. High-Intensity Interval Training Program

The HIIT program was supervised with the help of running and cycling ergometers, and exercise intensity was measured based on heart rate. Therefore, the formula maximum heart rate = 220-age was used to maintain the exercise heart rate at 85–100% [31]. 

The training time provided an acceleration time of 10 s until the target exercise intensity was reached, and maintained that intensity for 10–20 s. The wattage of the cycle ergometer and the speed and inclination of the treadmill were set through rehearsal training. The total recovery time and training time were 50–60 min. The detailed program has been outlined in Table 1. The LFG was performed twice a week, and the HFG was performed in five weekly sessions.

### 2.8. Statistical Analysis

The data analysis in this study was performed using IBM SPSS Ver. 25.0 (IBM Corp., Armonk, NY, USA). All variables were expressed as mean and standard deviation using descriptive statistics. Normality tests were performed using the Kolmogorov–Smirnov and Shapiro–Wilk tests. A non-parametric Wilcoxon signed-rank test was used to compare results pre and post the intervention. In addition, the Mann–Whitney U test was used for comparison between groups of LFG and HFG. All statistical significance levels were set at *p* < 0.05.

## 3. Result

Table 2 presents the characteristics. The two groups showed no significant differences in terms of age, height, weight, or body mass index. There was no significant difference in the proportion of injury sites and the duration of team departure due to injury. The proportion of knee injuries was the highest, followed by ankle injuries. The injuries history included knee, ankle, hip, and low back pain, and there was no difference between groups.

Table 3 compares the measurable variables in the GXT. As an effect of training, all variables significantly improved in both groups. Additionally, in terms of VO_2_peak, exercise duration, and heart rate recovery, the HFG showed a significantly higher in th post results than the LFG. However, there were no significant differences between groups in anaerobic threshold and ATHR.

Table 4 shows the results of an anaerobic power test using a bicycle ergometer. In the recorded 1st and 3rd set, the peak power and fatigue index improved post-test rather than pre- in both groups; however, only in HFG in 5th set. There was no significant difference of peak power between the two groups in the first set. In the third set, both groups showed improvement; however, there was a further improvement in HFG. In the 5th set, the peak power and fatigue index improved only in HFG but not in LFG.

Table 5 shows the results of the isokinetic muscle strength measurement test. At the angular velocity of 60°/s and 180°/s, measured for muscle strength and power, both groups showed improvement after the intervention compared to before the intervention; however, there was no significant difference between groups in post values. At 240°/s, where muscular endurance was measured, there was no significant improvement in the LFG post-test compared to the pre-test, and there was a significant change in the HFG. Therefore, we concluded that the interaction effect was apparent only at 240°/s.

In addition, Table 5 shows the changes in body composition pre and post training. Both groups showed a slight decrease in body fat and a slight increase in muscle mass; however, there was no significant difference between the groups.

## 4. Discussion

Although soccer requires a high level of physical fitness, it has a high injury rate because it is an active sport. Many players have to temporarily leave team training for rehabilitation or recovery due to injuries [1]. During the detraining period, athletes experienced a decrease in their physical fitness. However, depending on the athlete’s situation, it is sometimes difficult to visit the rehabilitation center every day, and it is not easy for high school students to visit the center frequently due to their academic commitments. Therefore, this study investigated the significance of the effect of low-frequency HIIT training over a short period.

In this study, the training effect was significant for other GXT variables, including VO_2_peak in both the HFG and LFG. In addition, there was a significant improvement, albeit in a short period. These results have also been reported in the literature. In a meta-analysis of 53 HIIT studies, the group that measured VO_2_peak showed a greater effect in the mid- to long-term but a significant effect even in a short period of fewer than 4 weeks. In the same study, even a short interval of less than 30 s and a low volume of less than 5 min showed an improvement [24]. Even in well-trained athletes, in the HIIT study conducted for 3 weeks, VO_2_peak showed a positive effect compared to the control group, and a positive training effect was found in groups where the maximum heart rate was decreased at the same exercise intensity [23]. Even short-term HIIT of six sessions for 2 weeks reported a greater increase in total work and session duration than MICT in citrate synthase maximal activity and mitochondrial respiration in skeletal muscle [32]. However, it was the lactate threshold in cases with no interaction between the two groups. The lactate threshold is one of the indicators used to assess the performance of athletes and is the primary purpose of HIIT training in particular. It was also reported that short-term training for three weeks did not show any significant improvement compared to the control group [33], although short-term training not only improved endurance but also had a significant effect on anaerobic power in trained athletes [34].

Soccer mainly relies on aerobic metabolism due to the long game time; however, activities that require explosive power, such as sprints, jumps, duels, and kicking, that use instantaneous power, have a decisive influence on the match results. Thus, it was found that good anaerobic athletic performance affected competition [6,35]. The Wingate test is a representative method for measuring the anaerobic power capability, and short-term HIIT induced positive changes. In short-term study, the peak power was improved by 16.7% for the upper body ergometer test and 8.5% for the lower body ergometer test through two sessions per week for 4 weeks [36]. In this study, the results of anaerobic power using the Wingate test showed improvement in sets 1st and 3rd of LFG but did not change in 5th. However, HFG showed a significant change in peak power even in the 5th. Similar results have been reported in previous studies. In the HIIT study conducted on badminton players, HIIT training three sessions per week for four weeks, totaling 12 sessions, improved peak power from one to four sets, but did not change in the 5th set [37]. This also means that HFG has a slightly better anaerobic power improvement effect than LFG.

A soccer player’s quadriceps muscles affect jumping and ball-kicking as well as running abilities, and the hamstrings act as antagonists when quadriceps power occurs, controlling posture and stabilizing the knee joint during turns or tackles [38,39]. The results of the isokinetic knee strength assessment in this study with a peak torque of 60°/s and an average power of 180°/s showed significant improvement in both groups, and the total work at 240°/s was significantly improved only in HFG. Although there was no change in muscle mass, the improvement in muscle strength seems to be due to the adaptation of nerves through improvement in muscle activity through training [40]. Another possible explanation is that improved muscle strength after HIIT may result from improved molecular signaling pathways, such as mitochondrial production and the ability to transport and oxidize carbohydrates and fats [41]. Nevertheless, there was no significant change at 240°/s possibly due to the focus on short-term power and muscle strength rather than endurance due to the nature of HIIT. This needs to be clarified in more detail in subsequent studies.

The body composition in this study showed a slight decrease in body fat and a slight increase in muscle mass; however, this change was not significant. This may be because the HIIT in this study was designed for short-term physical fitness improvement, and diet provision and hypertrophy training were not designed. Therefore, it was found that 4 weeks duration was not sufficient to change body composition, regardless of HFG or LFG. A previous study reported no significant change in body fat and muscle mass compared to the control group when HIIT training was performed for a total of nine sessions three times a week for 3 weeks [34]. In another study, despite performing HIIT for 12 weeks, there was an improvement in cardiorespiratory fitness but no significant change in muscle mass [42]. In a larger study, HIIT was performed in 138 judo, 40 taekwondo, 18 boxing, 17 karate, and 15 wrestling athletes. The VO_2_peak increased from 4.4 to 23%, but it did not cause any change in body fat percentage or body mass. [22]. In the case of well-trained athletes, there is a high possibility that they have low body fat and high muscle mass because they usually train frequently. Changing the body composition of athletes through short-term training is a challenging task, and short-term HIIT can be an effective training method in terms of time efficiency. However, when the goal is to improve body composition, it should be implemented with a long-term goal and plan.

HIIT effectively improves vertical and horizontal jumping ability, direction change, and aerobic ability in young soccer players [14]. In addition, HIIT training for athletes in other sports improves the athletic performance of swimmers and cyclists [10]. However, training professionals should be mindful that long-term daily HIIT training carries a potential risk of damage from overload. Prolonged HIIT eventually increases the likelihood of problems such as chronic pain syndrome and overuse syndrome, potentially leading to departure from team training [43]. Therefore, Smith et al. mentioned HIIT as an effective short-term strategy [34].

HIIT typically explains the mechanism for improving muscle adaptability despite a short exercise time as follows. HIIT can induce activation of AMP-activated protein kinase, peroxisome-proliferator-activated receptor-γ coactivator-1, Sirtuin 1 and reactive oxygen species pathways. Besides, it can enhance mitochondrial biogenesis and angiogenesis through regulation of Ca^2+^ homeostasis [44].

This study had a few limitations. This study was conducted on youth male soccer players. It is worth experimenting to see if the same results are obtained in other sports and female players. In particular, it will be interesting to compare the effects of HIIT training for athletes involved in winter sports, such as skiing, with athletes participating in summer sports. Although there are positive results of HIIT training on skiers [45,46], whether running or bicycle-based HIIT translates into good performance in skiing with a sliding pattern needs further examination. Moreover, it remains unknown how long the HIIT effect lasts after 4 weeks of training. In addition, to prevent the effect of accumulating muscle fatigue, it is necessary to be careful to perform the continuous high-intensity physical activity within 36 to 48 h [47]. Therefore, in the case of HFG in this study, sufficient recovery may not have been achieved. Finally, this study did not provide energy intake and nutritional management programs to youth athletes despite analyzing body fat and muscle mass. Although HIIT still has positive training effects for youth athletes, some researchers note that it does not have clear superiority compared to other types of training [9,15]. This is why future studies should be more concerned about effective fitness improvement training while avoiding the potential overload damage caused by HIIT in growing adolescents. 

## 5. Conclusions

High- and low-frequency HIIT was performed for youth soccer players who needed to improve their fitness for four weeks in their last stage of rehabilitation. HIIT was found to improve VO_2_peak, AT, anaerobic power, muscle strength, and muscle power in the LFG and HFG. Moreover, in terms of VO_2_peak, exercise time, 1 min recovery heart rate, anaerobic power, and muscular endurance, the results in HFG were more encouraging than LFG. However, there were limitations to changing body composition in both the HFG and LFG. Despite the above findings, we believe low-frequency HIIT will be an appropriate training method to improve physical fitness for youth soccer players with academic commitments, which makes participating in frequent HIIT sessions difficult.

## Figures and Tables

**Table 1:** High intensity interval training program.

Training Type	Bout	Intensity	Acceleration	Target Intensity Maintain	Interval Recovery	Set
Cycle ergometer	First bout	HRmax 85%	10 s	20 s	120 s	3
		Bout recovery 3 min				
	Second bout	HRmax 90%	10 s	15 s	120 s	3
		Bout recovery 3 min				
	Third bout	HRmax 100%	10 s	10 s	120 s	3
		End of cycle ergometer training and recovery 10 min				
Treadmill	First bout	HRmax 85%	10 s	20 s	120 s	3
		Bout recovery 3 min				
	Second bout	HRmax 90%	10 s	15 s	120 s	3
		Bout recovery 3 min				
	Third bout	HRmax 100%	10 s	10 s	120 s	3
		End of training				

Abbreviations: HRmax, maximum of heart rate.

**Table 2:** General Characteristics of participants.

Variables	LFG (*n* = 27)	HFG (*n* = 27)	*t*-Value	*p*-Value
Age, years	15.7 ± 0.8	15.8 ± 0.9	−0.242	0.710
Height, cm	176.4 ± 4.7	177.1 ± 4.3	−0.884	0.383
Weight, kg	63.8 ± 6.8	64.6 ± 7.1	−0.615	0.754
BMI, kg/m^2^	20.5 ± 1.8	20.6 ± 1.9	0.239	0.813
Injury duration, week	6.8 ± 1.2	7.0 ± 1.1	0.247	0.510
Injury site, *n*				
Ankle	5	8	0.450	0.258
Knee	17	17		
Hip	3	1		
Low back	2	1		

Abbreviations: LFG, low-frequency group; HFG, high-frequency group; BMI, body mass index.

**Table 3:** Exercise Graded Test with Cardiorespiratory Fitness.

Variables	Group	Pre	Post	*p*-Value
VO_2_peak, mL/kg/min	LFG	48.7 ± 6.9	56.4 ± 8.9	0.003
	HFG	50.1 ± 7.3	57.1 ± 9.0	0.009
	*p*-value	0.241	0.035	
Anaerobic Threshold, %	LFG	63.1 ± 6.0	69.3 ± 6.8	0.028
	HFG	62.9 ± 6.8	71.7 ± 6.7	0.017
	*p*-value	0.419	0.159	
ATHR, bpm	LFG	149.1 ± 12.3	158.0 ± 11.9	0.014
	HFG	148.9 ± 11.8	160.9 ± 12.1	0.002
	*p*-value	0.543	0.215	
Exercise duration, s	LFG	907.1 ± 32.1	972.3 ± 29.6	0.036
	HFG	918.7 ± 28.9	990.4 ± 30.1	0.025
	*p*-value	0.144	0.041	
Recovery 1 m HR, %	LFG	58.6 ± 8.8	62.3 ± 10.7	0.014
	HFG	57.7 ± 8.1	60.1 ± 12.1	0.011
	*p*-value	0.296	0.028	

*p* < 0.05; Abbreviations: LFG, low-frequency group; HFG, high-frequency group; AT, anaerobic threshold; ATHR, anaerobic threshold heart rate; HR, heart rate; bpm, beats per minute.

**Table 4:** Anaerobic test with Wingate cycle ergometer.

Set	Variables	Group	Pre	Post	*p*-Value
1 set	Peak power, watt	LFG	667.1 ± 110.7	766.6 ± 137.8	0.005
		HFG	680.9 ± 105.4	776.6 ± 127.0	0.014
		*p*-value	0.210	0.585	
	Peak power/kg	LFG	10.5 ± 2.1	11.9 ± 1.2	0.003
		HFG	11.0 ± 1.3	12.1 ± 1.3	0.015
		*p*-value	0.300	0.419	
	Fatigue Index	LFG	43.2 ± 14.8	35.8 ± 10.5	0.037
		HFG	45.4 ± 18.9	36.5 ± 10.9	0.008
		*p*-value	0.415	0.410	
3 set	Peak power, watt	LFG	636.4 ± 98.8	674.1 ± 76.7	0.019
		HFG	627.0 ± 88.9	685.2 ± 78.5	0.002
		*p*-value	0.516	0.021	
	Peak power/kg	LFG	10.0 ± 1.4	10.1 ± 1.2	0.039
		HFG	9.8 ± 1.5	10.9 ± 1.7	0.041
		*p*-value	0.410	0.025	
	Fatigue Index	LFG	51.6 ± 12.3	44.4 ± 13.1	0.017
		HFG	54.7 ± 10.5	39.5 ± 15.0	0.030
		*p*-value	0.315	0.017	
5 set	Peak power, watt	LFG	594.6 ± 81.4	606.3 ± 85.8	0.160
		HFG	603.1 ± 78.7	629.3 ± 81.6	0.019
		*p*-value	0.194	0.039	
	Peak power/kg	LFG	9.5 ± 1.6	9.6 ± 1.5	0.289
		HFG	9.3 ± 1.7	10.0 ± 1.4	0.024
		*p*-value	0.416	0.044	
	Fatigue Index	LFG	57.1 ± 10.6	56.1 ± 12.4	0.177
		HFG	59.1 ± 11.4	51.6 ± 12.6	0.013
		*p*-value	0.284	0.033	

*p* < 0.05; Abbreviations: LFG, low-frequency group; HFG, high-frequency group.

**Table 5:** Changes in isokinetic knee strength test and body composition.

	Variables	Group	Pre	Post	*p*-Value
Isokinetic knee strength	60°/s, peak Nm/kg, %	LFG	466.2 ± 58.9	482.7 ± 36.4	0.017
		HFG	464.9 ± 77.6	498.3 ± 64.5	0.002
		*p*-value	0.251	0.515	
	180°/s, average Watt/kg, %	LFG	613.4 ± 95.5	632.9 ± 53.3	0.017
		HFG	629.8 ± 98.8	647.8 ± 90.3	0.020
		*p*-value	0.274	0.610	
	240°/s, total Joule/kg, %	LFG	65.6 ± 9.8	68.5 ± 8.5	0.360
		HFG	64.4 ± 9.9	70.2 ± 10.5	0.006
		*p*-value	0.380	0.010	
Body composition	Fat mass, kg	LFG	7.4 ± 1.7	7.2 ± 1.7	0.805
		HFG	7.3 ± 1.5	7.1 ± 1.8	0.789
		*p*-value	0.332	0.250	
	Fat ratio, %	LFG	11.6 ± 2.1	11.1 ± 2.9	0.746
		HFG	11.4 ± 1.9	11.0 ± 2.5	0.527
		*p*-value	0.284	0.528	
	Muscle mass, kg	LFG	31.7 ± 4.7	32.3 ± 4.4	0.613
		HFG	31.7 ± 4.6	32.0 ± 4.2	0.245
		*p*-value	0.468	0.601	
	Muscle ratio, %	LFG	49.7 ± 2.1	50.1 ± 2.4	0.585
		HFG	50.1 ± 2.2	50.3 ± 2.4	0.628
		*p*-value	0.601	0.784	

*p* < 0.05; Abbreviations: LFG, low-frequency group; HFG, high-frequency group.

## Data Availability

The data are not publicly available because of privacy or ethics.
